# The Antidepressant-Like Effects of Shen Yuan in a Chronic Unpredictable Mild Stress Rat Model

**DOI:** 10.3389/fpsyt.2021.622204

**Published:** 2021-01-28

**Authors:** Ning Jiang, Haixia Wang, Hong Huang, Jingwei Lv, Guirong Zeng, Qiong Wang, Yu Bao, Ying Chen, Xinmin Liu

**Affiliations:** ^1^Research Center for Pharmacology and Toxicology, Institute of Medicinal Plant Development (IMPLAD), Chinese Academy of Medical Sciences and Peking Union Medical College, Beijing, China; ^2^Affiliated Traditional Chinese Medicine Hospital/School of Pharmacy/Sino-Portugal TCM International Cooperation Center, Southwest Medical University, Luzhou, China; ^3^Institute of Chinese Materia Medical, China Academy of Chinese Medical Sciences, Beijing, China

**Keywords:** SY, depression, CUMS, neurotransmitters, oxidative stress

## Abstract

Depression is a common yet severe neuropsychiatric condition that causes imposes considerable personal, economic, and social burdens worldwide. Medicinal plant species (e.g., *Panax ginseng* and *Polygala tenuifolia*) demonstrate potent antidepressant-like effects with less toxicity and other side effects. Shen yuan prescription (SY), composed of *Panax ginseng* (GT) and *Polygala tenuifolia* (YT). The present study aimed to elucidate the effects of SY treatment on chronic unpredictable mild stress (CUMS) rats and study the underlying mechanism. Our results indicated that SY (67.5, 135, or 270 mg/kg) significantly reverses the depressive-like behaviors in rats with a 5-week CUMS exposure, as demonstrated by increased sucrose consumption in the sucrose preference test, and decreased immobility time in the tail suspension and forced swim test. Moreover, SY altered serum corticosterone levels, pro-inflammatory cytokines (IL-6, IL-1β, and TNF-α), and oxidative markers (SOD, CAT, and MDA), and increased the levels of hippocampal neurotransmitters (5-HT, DA, and NE) in rats exposed to CUMS. Furthermore, rats treated with SY showed a reduction in the protein expression of BDNF, p-TrkB, p-Akt, and p-mTOR proteins induced by CUMS exposure in the hippocampus. In conclusion, SY prevented depressive-like behaviors in CUMS-exposed rats by preventing hypothalamus-pituitary-adrenal axis dysfunction, decreasing the levels of the neurotransmitters, minimizing oxidative stress, suppressing neuroinflammation, and activating the PI3K/Akt/mTOR-mediated BDNF/TrkB pathway, all of which are the key players in the pathological basis of depression.

## Introduction

The onset of depression, a neuropsychiatric disease, inflicts a significant global socioeconomic burden. With the ever-increasing pace of modern life, people experience increased stress in their professional and personal lives. The incidence of depression has increased throughout these years (1, 2). However, the pathogenic mechanisms underlying the onset of depression are unknown. Mounting evidence suggests that depression results from the dysfunction of the hypothalamus-pituitary-adrenal (HPA) axis, reduced secretion of neurotransmitters (such as 5-hydroxy tryptophan, norepinephrine, and dopamine), neuro-inflammation, oxido-nitrosative stress, reduced cell proliferation, abnormal cytokine secretion, depleted levels of neurotrophic factors, and disordered neuroplasticity (3–5). At present, antidepressants such as selective serotonin reuptake inhibitors mainly target monoamine levels; however, these drugs have side effects and high failure rates (6). Thus, antidepressants with increased effectiveness and acceptable safety profiles should be developed.

In recent years, the search for effective low-toxicity antidepressants, mostly from natural products and underlying mechanisms, is a promising research area. *Panax ginseng* (GT) and *Polygala tenuifolia* (YT), the traditional Chinese herbal medicines, possess a broad spectrum of neurotrophic and neuroprotective effects and are widely used for treating several neurological disorders, including depression (7, 8). In one of our earlier studies, we demonstrated the superior antidepressant efficacy of SY prescription compared with GT or YT alone using tail suspension and forced swim tests (9). Recent studies have indicated that SY upregulates hippocampal BDNF and TrkB expression and induces antidepressant-like effects in learned helplessness (LH) and chronic mild stress rat models (10, 11). In the present study, we utilized the chronic unpredictable mild stress (CUMS)-induced depression animal model to explore SY's antidepressant effects using the sucrose preference test, tail suspension test, and forced swimming test. We also measured the neurotransmitters and corticosterone levels, inflammatory mediators, and oxidative stress levels and studied the PI3K/Akt/mTOR-mediated BDNF/TrkB pathway to elucidate the underlying mechanism of action.

## Materials and Methods

### Chemicals and Reagents

Fluoxetine HCl (>98%) was procured from Aladdin Biochemical Technology Co., Ltd. (Shanghai, China). Norepinephrine (NE, noradrenaline) was purchased from the National Institute for Food and Drug Control (Beijing, China); dopamine (DA), 5-hydroxy tryptamine (5-HT) and DOPAC (dihydroxy-phenyl acetic acid) were purchased from Sigma-Aldrich Co. (St. Louis, MO, USA); and commercial kits for CORT, IL-6, IL-1β, TNF-α, superoxide dismutase (SOD), (LPO) and malondialdehyde (MDA) were from Jiancheng Biological Technology Co., Ltd. (Nanjing, China).

### SY Preparation

**Figure 1:**
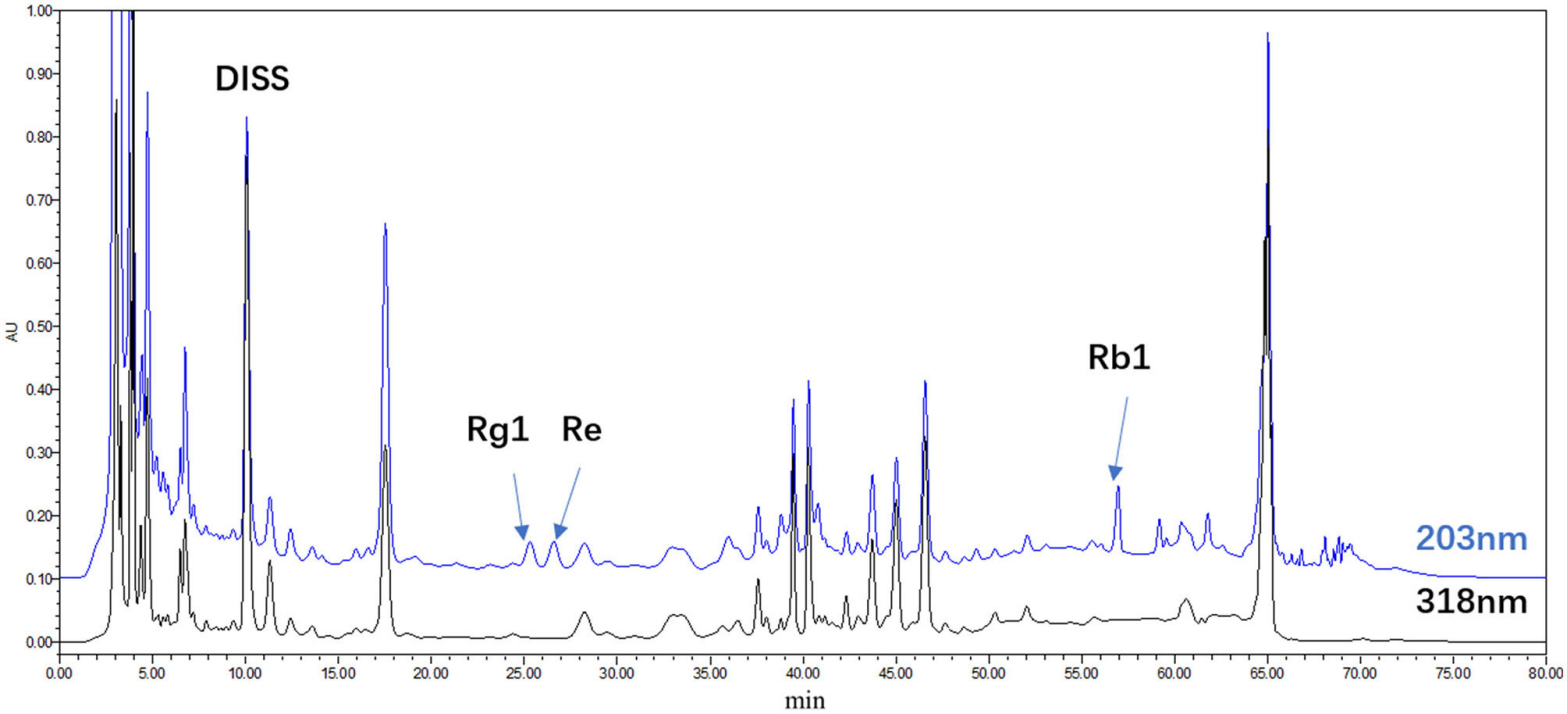
HPLC chromatogram of the water extract of *Panax ginseng* and *Polygala tenuifolia* (SY); DISS; Rg1; Re; Rb1.

Ginseng was purchased from the Jilin Shennong Traditional Chinese Medicine Technology Development Co., Ltd. (Jilin, China), and *Polygala tenuifolia* Willd was purchased from Shanxi Yuncheng Tiandi Net Chinese Herbal Medicine Co., Ltd (Shanxi, China). SY was prepared by water extracts from the stems of *P. tenuifolia* and *P. ginseng*. Briefly, dried roots of *P. tenuifolia* and *P. ginseng* (crude drug quantity ratio, 3:2) were extracted three times with deionized water, each time for 1 h, and the solid-liquid ratio was 8, 6, and 6, respectively. The extract was filtered through a 100-mesh sieve and combined. The filtrate was concentrated to a relative density of 1.125 (1.10–1.15, 25°C), followed by alcohol precipitation [concentration at 70% (w/w)], and refrigerated for 12 h at 10°C. Finally, after filtration by a 200-mesh sieve, the filtrate was evaporated and concentrated by spray drying (air inlet temperature was 180°C, air outlet temperature was 90°C) to obtain dry extract powder, and the paste extraction rate relative to crude drug was 26.4–27%. The refined anhydrous SY contained 0.26% 3',6-disinapoyl sucrose (DISS), 0.12% ginsenoside Rg1 (Rg1), 0.12% ginsenoside Re (Re), and 0.30% ginsenoside Rb1 (Rb1), as determined by HPLC on an anhydrous basis (Figure 1).

### Animals and Treatments

Sixty Sprague-Dawley (SD) rats (male; 180–200 g) (Institute of the Chinese Academy of Medical Science Center, Beijing, China) were housed under standard experimental conditions (20–22°C, 55% humidity, food and water *ad libitum*, 12:12 h light/dark cycle) and acclimatized. The animals had unrestricted access to food and water and were acclimatized to these conditions for 7 days. The animal ethics committee of the Institute of Medicinal Plant Development, Peking Union Medical College, sanctioned all animal experiments (approval no. SYXK 2017-0020).

**Figure 2:**
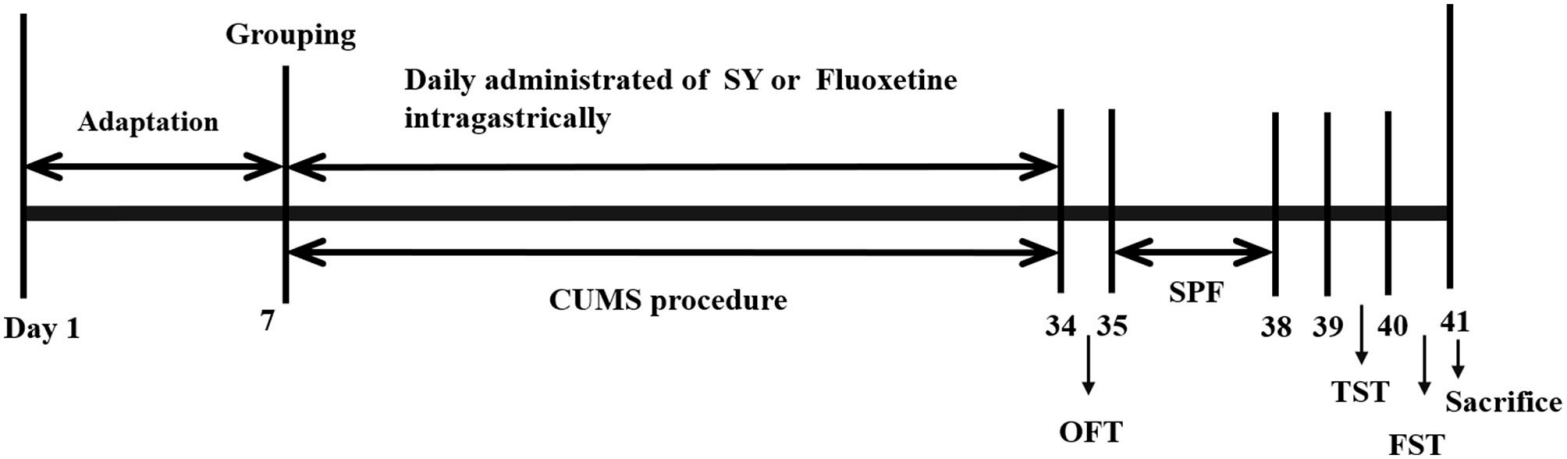
Experimental design procedure.

Animals were randomly divided into six groups: the control group, the CUMS model group, the SY-treated groups (67.5, 135, and 270 mg/kg) and the fluoxetine group (10 mg/kg). Different doses of SY or fluoxetine were administered orally to CUMS rats for 35 consecutive days until the behavioral tests were completed. The experimental procedure is illustrated in Figure 2.

### Chronic Unpredictable Mild Stress Model

The CUMS model was established following a modified procedure (9). The five test groups (excluding the control group) of rats were housed in individual cages and exposed to these stressors for 35 d: 24 h deprivation of food/water, 6 h restraint, 12 h illumination, 24 h light/dark alterations, 3 min tail pinch, predator sounds for 30 min, cold water (4°C) swimming for 5 min, electrical stimulation for 3 min, and hot water (40°C) swimming for 5 min. Rats were exposed to one or two different stressors daily for 5 weeks.

### Behavioral Tests

#### Open Field Test (OFT)

We used a self-developed computer-aided controlling system to assess the locomotive activity of the experimental rats on day 34 via OFT (12). The system constituted round metal pools (d × h: 75 cm × 40 cm) with a video camera fixed at the top, and four rats were placed at the center to explore freely for 5 min in light condition at 100 lux.

#### Sucrose Preference Test (SPT)

Post-CUMS period, we performed the SPT following previously described method with slight modification (13, 14). All rats were housed in individual cages for a 2 day adaptation phase and could drink from two bottles *ad libitum* (first day: two 1% sucrose solutions; the second day: 1% sucrose solution or tap water). Next, SPT was performed post-24 h food/water deprivation. Every rat was allowed to drink the pre-weighed sugar water and pure water for 1 h in a quiet and peaceful environment. Then, all the bottles were removed, weighed, and recorded. The following equation was used to calculate the sucrose preference: sucrose preference index (%) = (sucrose solution consumed/total solution consumed) × 100%.

#### Tail Suspension Test

The TST was performed following a previously described method (15, 16). Each rat was hung from a ledge for 6 min by the tail ~70 cm above the floor using adhesive tape. The “despair behavior” was recorded as the period of immobility during the total 6 min test duration.

#### Forced Swimming Test

The FST was conducted following a previously described method (11, 17). On the first day, each rat was constrained to swim for 15 min at a depth of 30 cm in an acrylic cylinder (d × h: 18 × 40 cm) at a temperature of 25°C). After 24 h, we reintroduced the rats into the same cylinder for a 5 min swimming test. The total period of immobility during the 5 min test duration was noted.

### Biochemical Analysis

#### Blood Sampling and Tissue Extraction

The rats were decapitated, and brain tissues were collected, followed by the isolation and weighing of the hippocampal tissue. After collection, blood was centrifuged (4°C; 15 min; 3,000 rpm). Both brain tissues and serum samples were stored at −80°C.

#### Measurement of the Neurotransmitter Levels in the Hippocampus

We used liquid chromatography-tandem mass spectrometry (LC-MS/MS) to determine the hippocampal levels of 5-HT, DA, and NE (18, 19). After homogenization, the hippocampal tissues were added to the acetonitrile solution containing 5 μg/mL of 3,4-dihydroxybenzylamine (DHBA, the internal standard) and centrifuged (4°C; 20,000 rpm; 30 min). Next, we measured the levels of the neurotransmitters in the supernatant via LC-MS/MS. We used a TSKgel Amide-80 column, maintained at 35°C. The separation was done at a flow rate of 0.4 mL/min using an 15 mM ammonium formate-acetonitrile solution in a 60:40 ratio (pH 5.5). The system was operated in the positive ion ESI mode under the multiple reaction monitoring (MRM) mode, at the m/z 177.0→ 160.0, 170.0→ 152.0, 154.2→ 136.6, 140.0→ 123.0 for 5-HT, NE, DA, and DHBA, respectively. The ratio of the peak areas of the analyte and DHBA were used to determine the concentration of the neurotransmitters.

#### Determination of Serum Corticosterone, Pro-inflammatory Cytokines, and Oxidative Stress Levels

The serum levels of IL-1β, IL-6, and TNF-α (Pro-inflammatory markers); catalase (CAT), superoxide dismutase (SOD), malondialdehyde (MDA), and corticosterone (CORT) (oxidative stress markers) were determined using commercial ELISA kits (Jiancheng, Nanjing, China).

#### Western Blot

After homogenization in RIPA lysis buffer (with protease/phosphatase inhibitors), the hippocampal homogenate was centrifuged (4°C; 12,000 g; 15 min), and quantified via the BCA protein assay. Next, the samples (30 μg) were electrophoresed (10% SDS-PAGE), and then transferred onto a PVDF membrane (Millipore, USA). The non-specific sites on the membrane were blocked by incubation for 1 h in 5% non-fat dry milk in TBS-T. Next, the membranes were kept in overnight incubation at 4°C with primary antibodies, including mTOR (1:1000, Cell Signaling); phospho-Akt (1:2000, Cell Signaling); phospho-TrkB (1:1000, Abcam); phospho-mTOR (1:1000, Cell Signaling); BDNF (1:5000, Abcam), and β-actin (1:1000, Cell Signaling). After washing, the membrane was incubated for 1 h with HRP-conjugated secondary antibody at room temperature. The enhanced chemiluminescence method was used to visualize the bands, which were captured using ChemiDoc XRS (Bio-Rad, USA). To eliminate variations in protein expression, three independent experiments were performed, and the data were adjusted to correspond to internal reference expression (β-actin), and the results are shown as a percentage of controls.

### Data and Statistical Analyses

All results were analyzed using SPSS Statistics 21.0 (SPSS Inc., Illinois, Chicago, USA). The differences between the means were analyzed by one-way analysis of variance (ANOVA) followed by Fisher's LSD *post hoc* test. Significance levels were considered at *p* < 0.05, and values are displayed as the mean ± standard error of the mean (SEM).

## Results

### Effects of SY on Depression-Like Symptoms in the CUMS Rat Model

**Figure 3:**
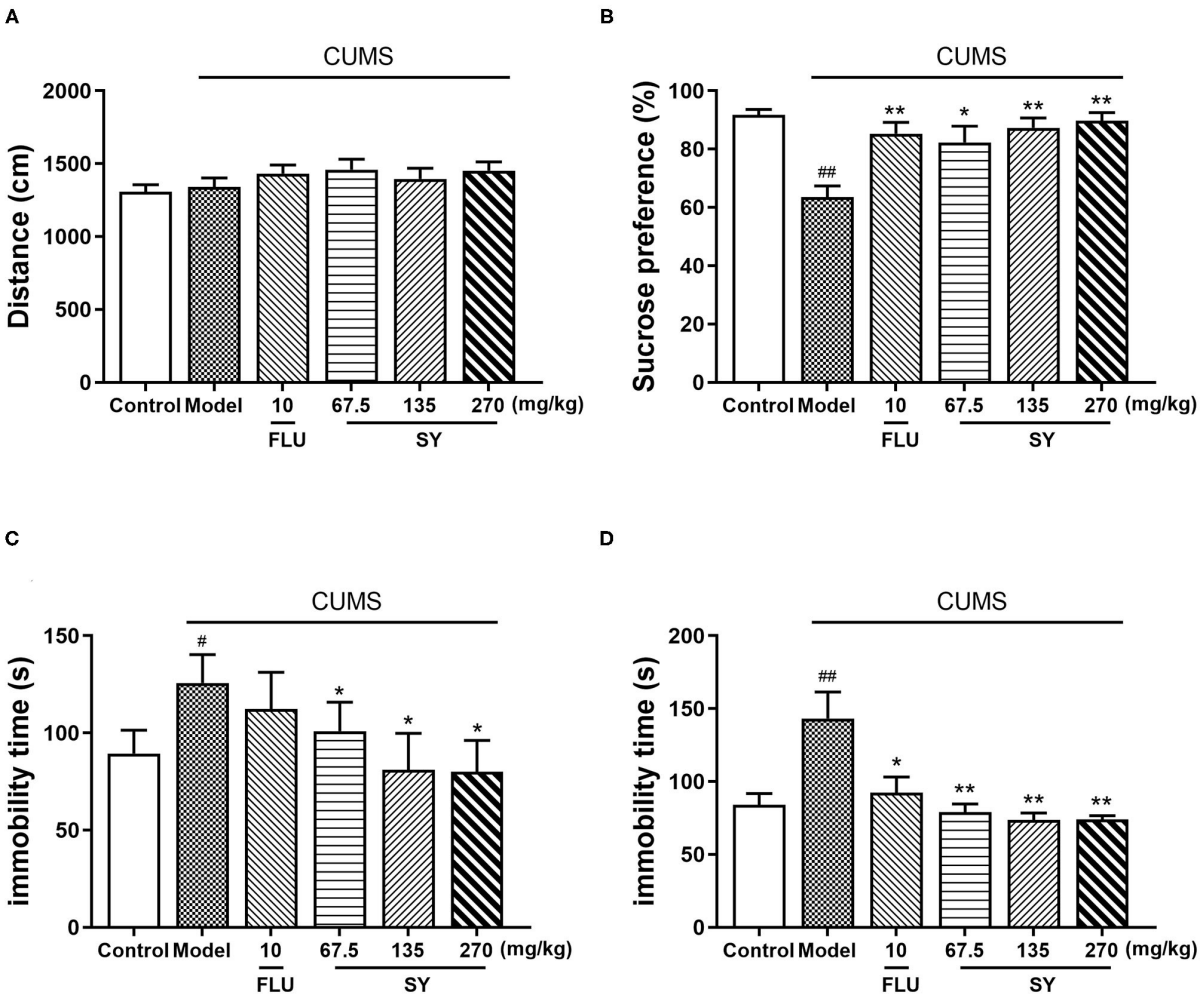
Antidepressant-like effects of chronic SY treatment on CUMS rats. **(A)** Open field test. **(B)** Sucrose preference test. **(C)** Tail suspension test. **(D)** Forced swim test. Data represent the mean ± S.E.M. (*n* = 10 per group). ^#^*p* < 0.05, ^##^*p* < 0.01, vs. the control group; *p < 0.05, **p < 0.01 vs. the CUMS group.

As per OFT results, we observed an insignificant difference in the total distance among all groups (*p* > 0.05; Figure 3A), which implies that SY treatment did not affect the locomotor activities of rats. Thus, the following behavioral tests could be used reliably to assess the animals.

Figure 3B shows the performance of rats in the sucrose preference test (SPT). Sucrose consumption was decreased in CUMS-exposed rats to the same extent as in the control rats [*F*_(5,54)_ = 4.606, *p* < 0.01]; the decreases in both the groups were significant. However, the decreases in sucrose consumption were considerably alleviated in all SY treatment groups (*p* < 0.05, *p* < 0.01, *p* < 0.01), consistent with the decrease in 10 mgkg^−1^ fluoxetine group (*p* < 0.01).

In the tail suspension test (TST), CUMS-exposed rats exhibited behavioral despair, as shown by substantially enhanced immobility time as compared with no-stress control rats [Figure 3C; *F*_(5,54)_ = 4.606, *p* < 0.01]. However, treatment with SY (67.5, 135, and 270 mgkg^−1^) considerably reduced the immobility time in CUMS-exposed rats, especially at 270 mgkg^−1^ (all *p* < 0.05).

Figure 3D shows the results of the forced swimming test (FST). We observed a substantial increase in the immobility time in the CUMS group as compared with the control group after a 5 week exposure to the stressors [*F*_(5,54)_ = 7.495, *p* < 0.01], which was considerably reversed after administration of SY (67.5, 135, and 270 mgkg^−1^) or fluoxetine (10 mgkg^−1^) (*p* < 0.01, *p* < 0.01, *p* < 0.01, and *p* < 0.05, respectively).

### Effects of SY on the Levels of Oxidative Stress Markers

**Figure 4:**
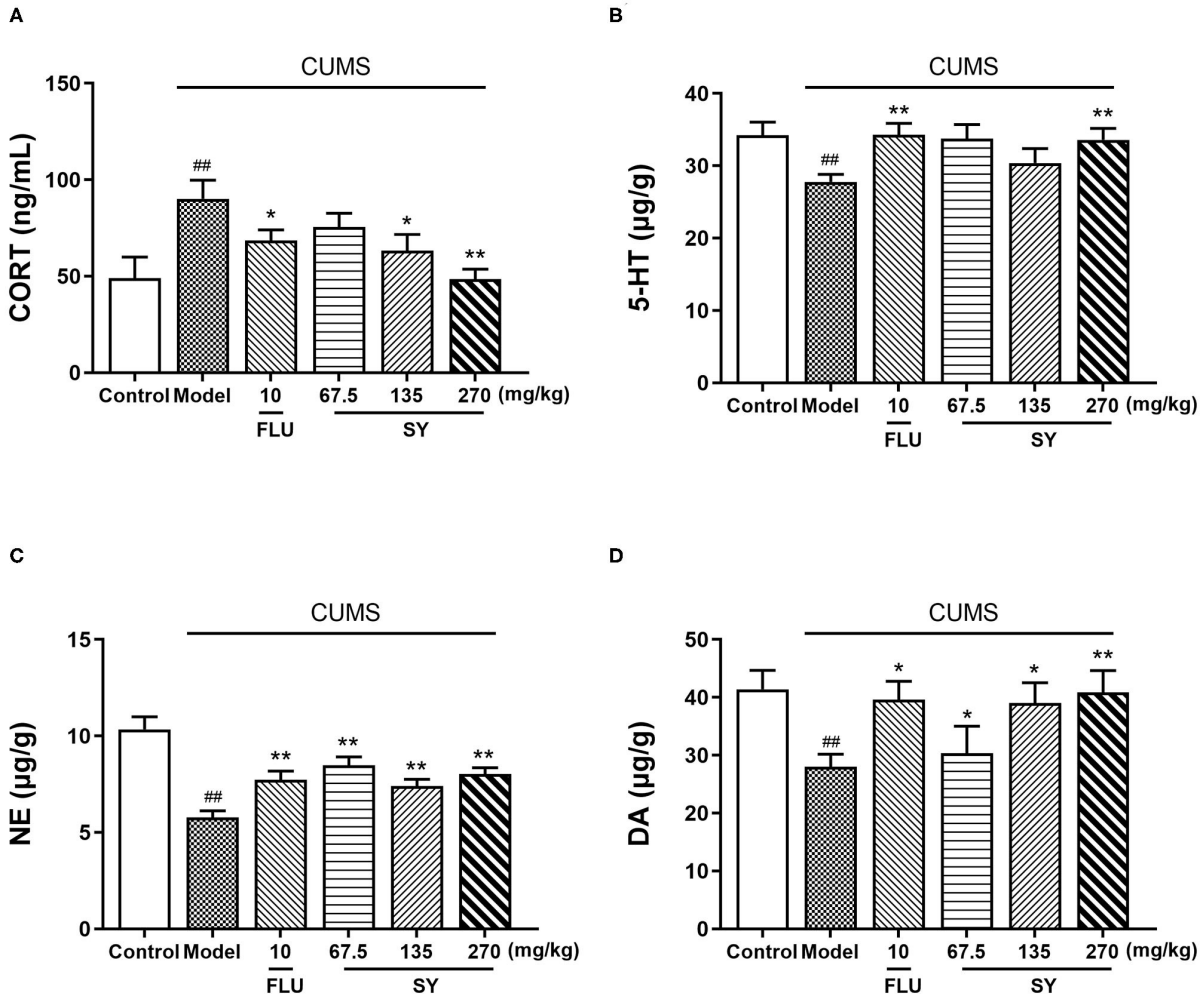
Effects of SY on the levels of CORT, 5-HT, NE, and DA in CUMS rats. **(A)** Levels of corticosterone (CORT). **(B)** Levels of 5-hydroxy tryptamine (5-HT). **(C)** Levels of norepinephrine (NE). **(D)** Levels of dopamine (DA) in each group. Data represent the mean ± S.E.M. (*n* = 10 per group). ^##^*p* < 0.01, vs. the control group; *p < 0.05, **p < 0.01 vs. the CUMS group.

The CUMS-exposed rats exhibited elevated levels of serum corticosterone (CORT) than the controls [Figure 4A; *F*_(5,54)_ = 5.987, *p* < 0.01]. However, 42 d treatment with SY (135 and 270 mgkg^−1^) or fluoxetine (10 mgkg^−1^) substantially reversed the CUMS-induced elevation of serum CORT levels (*p* < 0.05, each).

Figures 4B–D demonstrate a significant reduction in the hippocampal levels of NE, 5-HT, and DA post-CUMS induction as compared with the unstressed controls (*p* < 0.01 each). However, SY (270 mgkg^−1^) or fluoxetine (10 mgkg^−1^) considerably suppressed the decrease in the elevated levels of 5-HT [Figure 4C; *F*_(5,54)_ = 4.425, *p* < 0.01]. All SY-treated groups (67.5, 135, and 270 mgkg^−1^) exhibited significantly increases in NE [Figure 4C; *F*_(5,54)_ = 11.666, *p* < 0.01 each] and DA levels [Figure 4D; *F*_(5,54)_ = 4.220, *p* < 0.01, *p* < 0.01, and *p* < 0.05, respectively].

### Effects of SY on Serum Oxidative Stress Biomarkers and Pro-Inflammatory Levels

**Figure 5:**
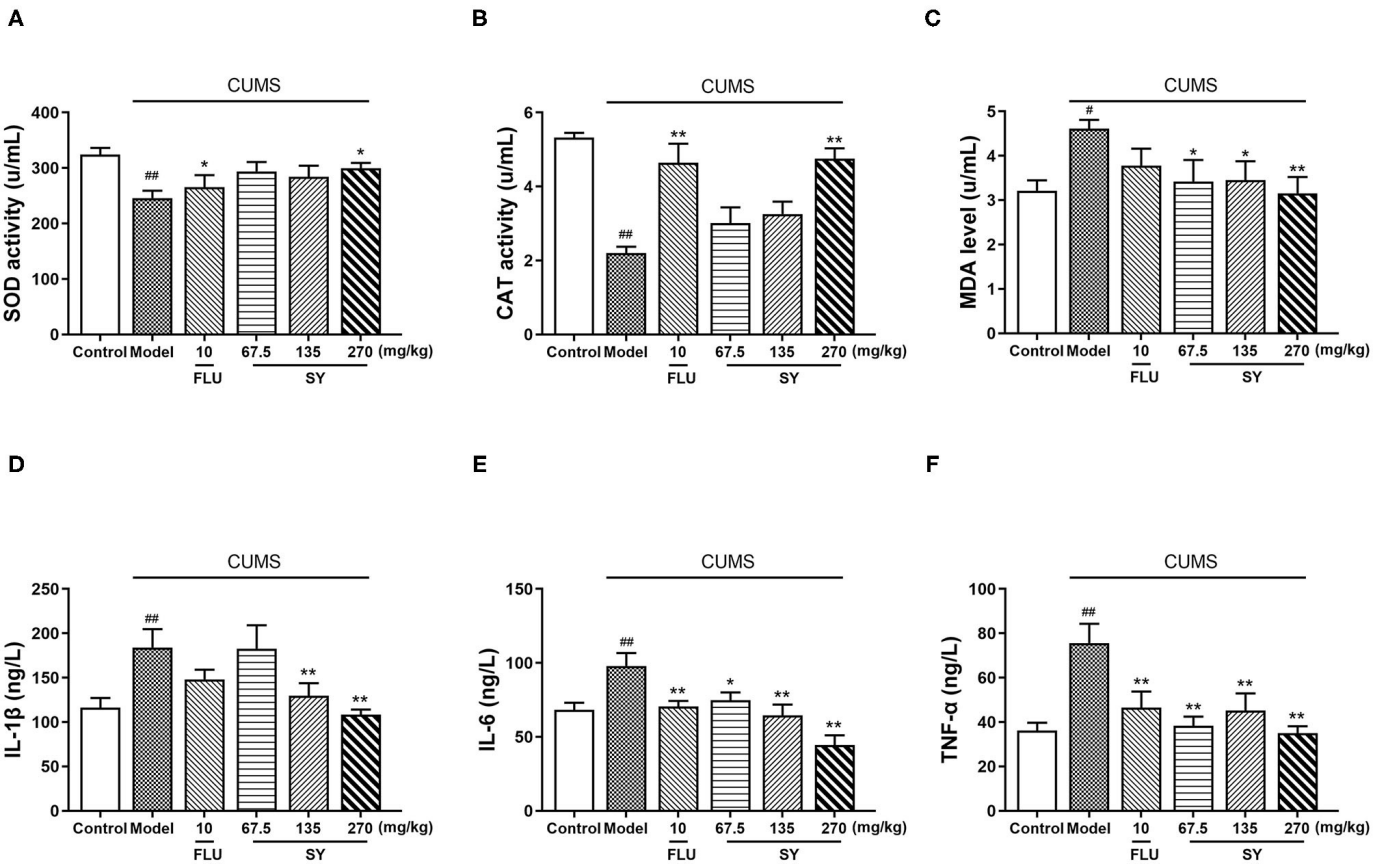
Effects of SY on oxidative stress biomarkers and pro-inflammatory cytokines in CUMS rats. **(A)** SOD activity. **(B)** CAT activity **(C)** MDA level. **(D)** IL-1β level. **(E)** IL-6 level. **(F)** TNF-α level. Data represent the mean ± S.E.M. (*n* = 10 per group). ^#^*p* < 0.05, ^##^*p* < 0.01, vs. the control group; *p < 0.05, **p < 0.01 vs. the CUMS group.

We measured the serum concentration of SOD, CAT, and MDA to assess the impact of the CUMS procedure and SY treatment on redox homeostasis. We observed a substantial reduction in SOD and CAT levels [Figures 5A–C; *F*_(5,54)_ = 2.851, *F*_(5,54)_ = 10.940, *F*_(5,54)_ = 2.218, *p* < 0.01 each] and a substantial increase in MDA levels (*p* < 0.01) in CUMS group as compared with the control group. Serum levels of SOD and CAT increased after treatment with SY (270 mgkg^−1^) (*p* < 0.05, *p* < 0.01); MDA levels decreased considerably post-treatment with SY (67.5, 135, and 270 mgkg^−1^) as compared with the CUMS group (Figure 4C; *p* < 0.05, *p* < 0.05, and *p* < 0.01, respectively).

Next, we estimated the hippocampal levels of IL-1β, IL-6, and TNF-α to confirm the protective response of SY on CUMS-induced neuroinflammation *in vivo*. CUMS procedure induced a substantial increase in the serum levels of IL-1β, IL-6, and TNF-α [Figures 5D–F; *F*_(5,54)_ = 3.948, *F*_(5,54)_ =2.294, *F*_(5,54)_ = 1.728, all *p* < 0.01]. Additionally, there was a significant decrease in serum IL-1β levels after treatment with SY (135 and 270 mgkg^−1^) in the CUMS group (Figure 4D; *p* < 0.05 and *p* < 0.01). Treatment with SY (67.5, 135, and 270 mgkg^−1^) significantly reversed the elevation in the serum levels of IL-6 and TNF-α (Figure 4E; *p* < 0.01, Figure 4F; *p* < 0.01, *p* < 0.01, and *p* < 0.05, respectively).

### Effects of SY on PI3K/Akt/mTOR-Mediated BDNF/TrkB Pathway-Related Protein Expression

**Figure 6:**
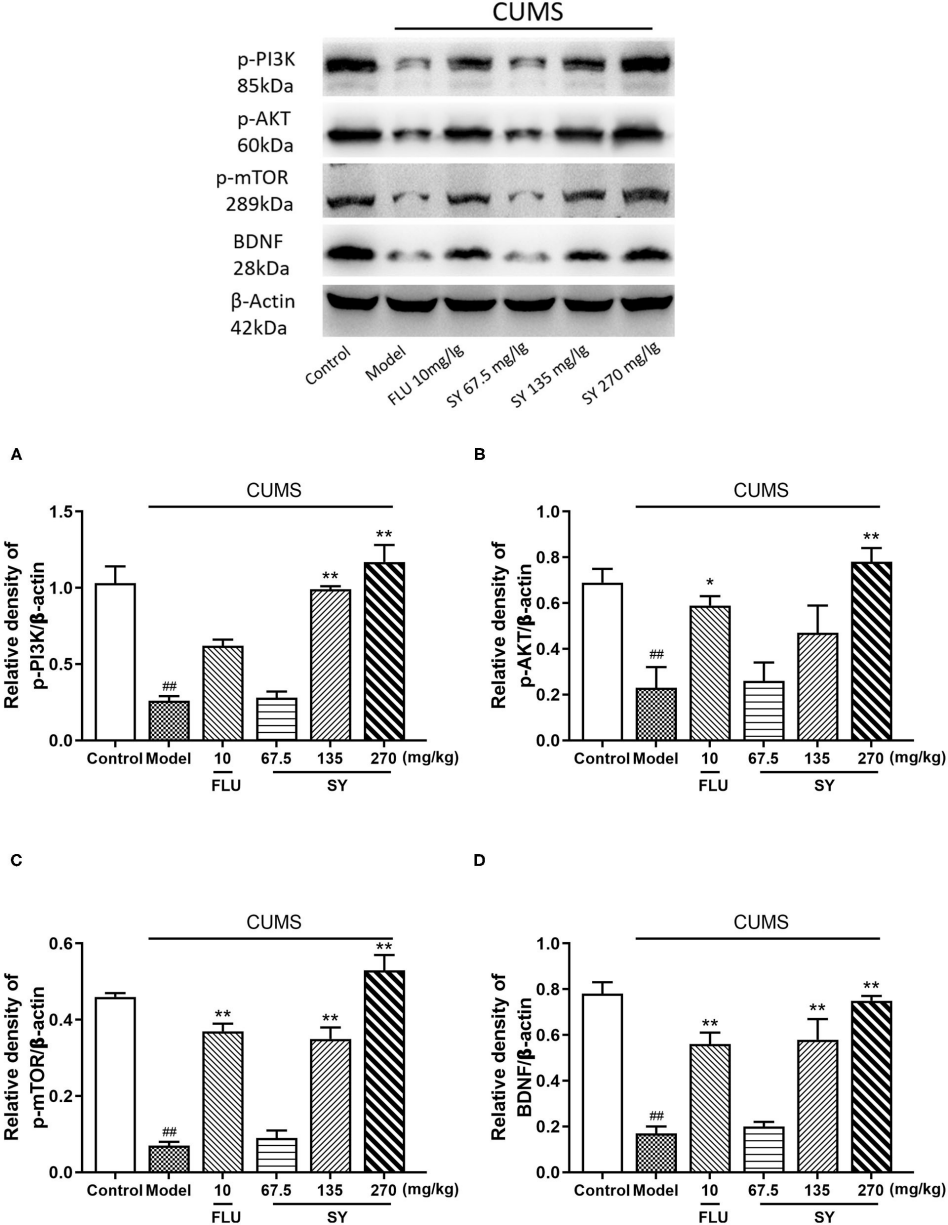
Effects of SY on the hippocampal expression of p-PI3K, p-AKT, p-mTOR, and BDNF in CUMS rats. **(A)** Levels of p-PI3K. **(B)** Levels of p-AKT. **(C)** Levels of p-mTOR. **(D)** Levels of BDNF in each group. Data represent the mean ± S.E.M. (*n* = 10 per group). ^##^*p* < 0.01, vs. the control group; *p < 0.05, **p < 0.01 vs. the CUMS group.

We further evaluated the expression of PI3K/Akt/mTOR-mediated BDNF/TrkB pathway-related proteins to investigate the role of SY in depression. The western blotting results (Figures 6A–D) indicated that CUMS exposure significantly induced a decrease in hippocampal phosphorylated PI3K, phosphorylated Akt, phosphorylated mTOR, and BDNF expression as compared with control rats (all *p* < 0.01). However, varying degrees of increases were observed in the expression of BDNF, phosphorylated PI3K, Akt, and mTOR in all the SY-treated groups (*p* < 0.01 each).

## Discussion

We observed that high concentrations of SY alleviated CUMS-induced depression symptoms, including anhedonia and despair, as demonstrated by significantly increased sucrose preference in SPT and reduced immobility in TST and FST in rats. At the same time, SY treatment significantly decreased the serum levels of CORT and increased hippocampal levels of neurotransmitters (NE, 5-HT, and DA) in CUMS-exposed rats. SY also alleviated oxidative stress and inhibited inflammation in CUMS-exposed rats, as evidenced by elevated SOD activity and CAT levels, lowered MAD content, and decreased IL-1β, IL-6, and TNF-α serum levels. Moreover, we found that SY-treated rats displayed enhanced hippocampal expression of BDNF, p-Akt, p-PI3K, and p-mTOR proteins.

Katz (20) observed that the rats showed reduced activity ability after getting exposed to a series of stress factors such as bright light stimulation, noise, and prolonged behavioral restriction. Willner (21) developed a CUMS depression model based on their observations. As compared with Katz's scheme, Willner's procedure had two changes: the reduced intensity of stimulants and the use of anhedonia as the measure of the model's success instead of the change in exercise ability. CUMS model simulates human beings' continuous acceptance of uncontrollable adverse events in social life and applies several different stress factors in random order throughout the experiment so that the animals cannot anticipate the occurrence of stimuli. As compared with the animal model of depression caused by acute stress such as forced swimming or electric shock, CUMS model can better simulate the social and environmental stress caused by depression, with higher credibility, and is the most important animal model for screening antidepressants and their mechanism of action (22). Accumulating evidence has demonstrated that a 5-week CUMS exposure can elicit depression-like symptoms, including anhedonia and despair behavior, as shown by reduced sucrose intake in the SPT and enhanced immobility time in the TST and FST (23), which is consistent with the current study. As compared with treatment with classical antidepressants (fluoxetine and imipramine), SY treatment was found to significantly reverse the depression-like behaviors, consistent with a previous report (11). Therefore, this study proved that SY possesses significant antidepressant activity.

When the organism is in a state of stress, the hypothalamic-pituitary-adrenal (HPA) axis will improve its excitability and promote the organism's effective adaptive regulation, which is conducive to preserving life and adaptation to the environment. The HPA axis eventually releases CORT or cortisol that acts on corticosteroid receptors in the brain and is involved in negative feedback regulation in the hypothalamus and pituitary gland. Serum CORT or cortisol levels can largely reflect the status of the HPA axis. However, under long-term stress, the HPA axis is continuously excited and may even damage its negative feedback regulation mechanism; thus, the entire HPA axis cannot be suppressed. Excessively high CORT or cortisol levels for a long time are implicated in the pathophysiology of depression. Depressed patients have significantly high cortisol concentrations, and depression model animals also show sharply rising corticosterone levels (24). In the present study, CUMS exposure caused a significant increase in rats' serum CORT levels, reflecting HPA axis hyperfunction, which is in line with previous studies. SY significantly decreased these elevated CORT levels, indicating that SY might elicit its antidepressant effects by inhibiting the hyperactivation of the HPA axis. The monoamine hypothesis put forward in the last century is the classical hypothesis of depression, which has been widely accepted and verified. This hypothesis suggests that depression results from a deficiency in monoamine neurotransmitters in the brain, and the clinical antidepressants currently in use target the monoaminergic system (25). CUMS procedure reduces hippocampal and prefrontal cortex levels of monoamines, including DA, 5-HT, and NE, inducing monoamine deficiency (18). Our results also showed that NE, 5-HT, and DA levels in CUMS-exposed rats were significantly lower than those of non-stressed control rats. As expected, the administration of SY significantly reversed this effect for 5-HT, DA, and NE and increased their secretion to levels similar to those in rats that were given the antidepressant Flu. These observations indicated that this normalization of neurotransmitter expression might induce the antidepressant effects of SY.

Increasing evidence supports the view that depression and chronic stress-induced depression-like behavior are associated with inflammation (26). Excessive secretion of IL-1β, IL-6, and TNF-α triggers depression-like symptoms. Indeed, depressive patients exhibit upregulated levels of pro-inflammatory cytokines (27). Animal studies have also shown that some depressive-like symptoms are related to the chronic stress-mediated secretion of inflammatory cytokines (28). Accordingly, rats subjected to CUMS exhibited elevated levels of IL-1β, IL-6, and TNF-α; however, SY treatment successfully reversed this effect. Inflammation is closely related to oxidative stress. Under normal circumstances, there is a dynamic balance between the body's antioxidant capacity and oxidative capacity. During oxidative stress, a large number of free radicals are generated, causing an imbalance in the body's oxidation-antioxidant system, leading to mitochondrial damage, NO release, and MDA accumulation (29). The level of cytokines is increased, and the activity of antioxidants (SOD, CAT, and GSH) is decreased, causing damage and apoptosis of hippocampal neurons in the brain. These manifestations induce depression or aggravate the course of depression (30, 31). Similar to previous studies, our study also reports that a 5-week exposure to CUMS causes substantially reduced SOD and CAT levels and a notable increase in the serum concentration of MDA in rats. However, SY (67.5, 130, and 270 mgkg^−1^) regulated the expression of these oxidative stress indicators in model rats to varying degrees and improved the depression-like symptoms. These findings suggest that SY exhibits its antidepressant effects by inhibiting immune-mediated inflammation and suppressing oxidative stress.

The hippocampus, which is a key brain area for learning, memory, and emotional disorders, is important in mediating the stress response and is also extremely vulnerable to damage, and it has been implicated in mood disorders (32). BDNF is one of the four major members of the neurotrophic factor family and is widely distributed in the mammalian brain. It is primarily expressed in the hippocampus and prefrontal cortex, among other areas. BDNF is vital for the pathogenesis of depression, cell plasticity regulation, inhibition of cell cascade death, increase of cell survival proteins related to neuron proliferation and maintenance, etc. Large evidence suggests that human and animal cases of depression exhibit downregulated hippocampal expression of BDNF (33). A variety of antidepressants (serotonin reuptake inhibitors and tricyclic antidepressants) can reverse the decrease in BDNF expression and significantly improve neuronal damage (7). Similar to other reports, we found that SY significantly improves depression-like symptoms and reduces CUMS-induced BDNF expression. This observation confirmed that SY exhibits its neuroprotective ability via the BDNF signaling pathway.

CUMS not only reduces BDNF expression but also results in significantly downregulated phosphorylation of Akt, PI3K, and mTOR in the hippocampus. These manifestations were reversed via SY treatment. The PI3K/Akt signaling pathway is the primary downstream signaling pathway in BDNF/TrkB signaling, regulating neuronal cell growth and survival in the hippocampus and mediating stress-induced depression and antidepressant effects (34, 35). Mammalian target of rapamycin (mTOR) is a downstream signaling molecule of PI3K/Akt pathway that regulates protein translation and synthesis. Depression is caused by synaptic protein defects induced by abnormal mTOR signaling (36). Recent studies have identified mTOR signaling as one of the targets involved in the rapid antidepressant response. Animal studies detected reduced phosphorylation of mTOR and Akt in the hippocampus of CUMS-exposed mice (37). Previous studies demonstrated that ketamine increases synaptic protein synthesis by activating the mTOR pathway, increases synaptic function, promotes synapse occurrence, and produces rapid antidepressant effects (38, 39). Similarly, the classical antidepressant fluoxetine modulates the mTOR signaling pathway in the hippocampus of mice exposed to chronically CUMS (40). These results suggest that the PI3K/Akt/mTOR signaling pathway plays an irreplaceable role in treating depression. We found that CUMS induced depression-like symptoms and reduced PI3K/Akt/mTOR phosphorylation in the hippocampus were ameliorated by long-term SY treatment. Overall, these neurochemical results suggest that SY upregulates the expression of BDNF/TrkB and the PI3K/Akt/mTOR signaling pathway to ameliorate CUMS-induced depression-like symptoms in rats.

Our study showed that SY prevents depression-like symptoms in CUMS-exposed rats by preventing HPA dysfunction, decreasing the neurotransmitter levels, minimizing oxidative stress, suppressing neuroinflammation, and activating the PI3K/Akt/mTOR-mediated BDNF/TrkB pathway, all of which are the key players in the pathological basis of depression. These findings suggest that SY can function as a potent therapeutic agent for preventing and treating stress-associated disorders, including depression.

## Data Availability Statement

The original contributions presented in the study are included in the article/supplementary material, further inquiries can be directed to the corresponding author/s.

## Ethics Statement

The animal study was reviewed and approved by Institute of Medicinal Plant Development, Peking Union Medical College, sanctioned all animal experiments (approval no. SYXK 2017-0020).

## Author Contributions

NJ, JL, and XL participated in the experiment design. NJ, HW, and HH conducted the experiments and performed the data analysis. NJ, JL, HW, HH, YB, GZ, and XL contributed to the writing and amendments of the manuscript. YC and QW were responsible for the supervision and project administration. All authors discussed, edited, and approved the final version.

## Conflict of Interest

The authors declare that the research was conducted in the absence of any commercial or financial relationships that could be construed as a potential conflict of interest.
